# Internet- and mobile-based anxiety and depression interventions for children and adolescents: efficacy and negative effects - a systematic review and meta-analysis

**DOI:** 10.1007/s00787-024-02404-y

**Published:** 2024-03-02

**Authors:** Patrick Dülsen, Harald Baumeister

**Affiliations:** Department of Clinical Psychology and Psychotherapy, Institute of Psychology and Education, Ulm University, Lise-Meitner-Straße 16, 89081 Ulm, Germany

**Keywords:** Internet intervention, Children and adolescents, Anxiety, Depression, Negative effects

## Abstract

Mental disorders, most commonly anxiety disorders and fourth most common depression, are prevalent in children and adolescents. Internet- and mobile-based interventions might represent a scalable approach to improve mental health care, however, evidence so far is inconclusive and systematic reports on negative effects are missing. Four data-bases were searched for randomized controlled trials evaluating internet- and mobile-based interventions (IMIs) targeting anxiety disorders or depression in children and adolescents up to 18 years exhibiting clinically relevant symptoms. Meta-analytic evaluations were conducted in comparison to active and passive control groups, furthermore, pre-defined sub-groups were explored and reported negative effects examined. Pooled estimates showed a moderate positive effect for IMIs targeting anxiety disorders compared to passive control groups (g = -0.69; CI -0.94 to -0.45; k = 8; *n* = 559; *p* ≤ 0,001), but not for depression. Pooled estimates compared to active control groups remained non-significant. Subgroup analyses were largely omitted due to an insufficient number of trials or were non-significant. Negative effects were mainly reported as drop-out rates and (non)-response rates, while additional negative effects, such as deterioration rates or the development of additional symptoms, were reported by only one third of included studies. The focus on children and adolescents with clinically relevant symptoms allowed the present findings to complement previous work, however, the limited amount of trials hindered many planned comparisons. The overview of reported negative effects highlighted that negative effects are being neglected in the majority of RCTs. Hence, in the future RCTs should include more information about potential negative effects, at best a combination of quantitative and qualitative information. Open Science Framework (osf.io/ch5nj).

**Supplementary Information:**

The online version contains supplementary material available at 10.1007/s00787-024-02404-y.

## Introduction

Mental disorders account for around 13% of the global burden of disease for children and adolescents between the ages of 10 and 19 [1]. The most prevalent mental disorders are anxiety disorders (3.6% of 10–14 years old, 4.6% of 15–19 years old) while depressive disorders regularly rank as forth most prevalent (1.1% of 10–14 years old, 2.8% of 15-19-year old) [1]. Research indicates that the onset of half of all mental disorders occurs during childhood or adolescents, however, treatment typically often starts only several years later [2–4]. Due to the fact that these early years in a person’s life are such a malleable and developmentally important period, it seems essential that young individuals receive appropriate treatment at the onset of mental disorders. Treatment delivered in time would allow children and adolescents to engage with the upcoming challenges, can improve several treatment outcomes and reduce mental disorders exhibited as adults [3].

Several obstacles for the treatment of mental disorders are present at the moment. There is, for example, a lack of mental health awareness, still much stigmatization around the topic of mental disorders and its treatments, a lack in financial resources and a limited availability of mental health care professionals as well as services [5–7]. The last point has often been described in the literature as mental health care gap, a gap between the limited amount of available treatment and the amount of people in need of it [8]. Such a gap often leads to longer waiting times before a treatment can be started.

To account for each individual obstacle different approaches have been tested. One approach is the use of internet- and mobile-based interventions (IMIs; [9]). Such interventions are often defined as self-help interventions which can be accessed through the internet via a web browser on computers/tablets and/or as apps on smartphones/tablets. Usually they are accompanied with some sort of human assistance and feedback is provided in an (a)synchronous fashion [9]. Thanks to their time, location and generally personal independent designs, IMIs are a scalable mental health care offer [9].

Apart from being scalable to a large group of people looking for treatment, IMIs have also been shown to be efficacious in treating a wide range of mental disorders in young individuals [10–16]. Some evidence in the literature even suggests comparable effectiveness for IMIs and face to face interventions for children and adolescents [10], while other findings contradict this [16]. However, as is often the case, the amount of evidence available for children and adolescents is still small compared to the evidence available for adults. Furthermore, the clear separation between samples of children and adolescents (below 18 years) and samples including young adults (up to 25 years) is often missing in the literature. This is especially interesting since several authors indicated a differing efficacy for individuals above and below 18 years [11, 16]. Additionally, available RCTs often use samples that combine participants with mild and severe symptom levels or with and without diagnosed disorders [11, 17] lacking a clear differentiation between these groups. However, such a level of differentiation might be necessary to further our understanding on potentially existing differences in efficacy of IMIs across age and mental health ranges.

Information that is also lacking, in both RCTs and systematic reviews, is reported negative effects [18]. Several attempts have been put on the way to establish common ways of defining, measuring and reporting negative effects in the psychotherapeutic literature [19–21]. Lately these efforts of establishing common ways have also been put forward for IMIs [22, 23]. It was suggested to differentiate between deterioration, adverse events, serious adverse events, novel symptoms, drop-out, non-response and unwanted events [22]. Systematic reviews that specifically evaluate if and how negative effects are being measured and reported in RCTs evaluating IMIs are not available at the moment. Only one systematic review about negative effects during psychotherapeutic treatments in general for all ages [18] and individual participant meta-analyses evaluating deterioration rates for adult samples [24–26] exist so far.

Correspondingly, the present systematic review and meta-analysis has three main research objectives. First, update current reviews [11–13, 27] regarding the available evidence for internet- and mobile based interventions for children and adolescents targeting depression and anxiety disorders. Thereby extending available reviews by focusing on children and adolescents instead of the broader concept of youth up to often 25 years of age [11, 12, 28] as well as only including samples with clinically relevant symptom levels, existing reviews often included mixed samples [11, 13, 28]. Second, evaluate if the exclusive focus on children and adolescents up to the age of 18 with clinically relevant symptoms has an impact on pre-defined subgroups. Third, examine reported negative effects, as no review focusing on children and adolescents has done this so far.

## Methods

The present systematic review and meta-analysis was registered at the Open Science Framework (osf.io/ch5nj) and is reported according to the PRISMA guidelines for meta-analyses [29].

### Eligibility criteria

Included studies had to (1) focus on children and adolescents (sample mean age ≤ 18), (2) with depression and/or anxiety symptoms on a clinically relevant level (as assessed by standardized diagnostic interviews, by applying an established cut-off score on a self-report scale or respective diagnosed disorders by a mental health professional). The reported interventions (3) had to be internet- and/or mobile-based interventions delivered via web-pages or via apps for smartphones or tablets, (4) be based on evidence-based backgrounds (e.g. cognitive behavior therapy (CBT), psychodynamic therapy, or acceptance and commitment therapy), and had to have (5) a mental health focus targeting depression and/or anxiety disorders, in a (6) guided or unguided (7) stand-alone fashion (no combination of online and offline interventions; group settings were excluded as well). The study design had to be (8) a randomized controlled trial (RCT) with various control conditions (i.e. wait-list control group or treatment as usual). Outcomes needed to (9) focus on depression and/or anxiety symptoms (i.e. self-report questionnaires or observer rated instruments). All included studies needed to (10) be published in English.

### Literature search

The Literature search was conducted in four major bibliographical databases, Embase, PubMed, PsycInfo as well as Cochrane controlled trial register (CENTRAL) and included all publications until the 7th of June 2022. A general search string was individually adapted to the specifications of each database accessed through Ovid. Furthermore, reference lists of included studies were manually screened for additional not yet included studies.

### Study selection and data extraction

In a first step, one reviewer (PD) screened all studies and excluded those that clearly did not fit the eligibility criteria based on their titles and abstracts. During the second step, two reviewers (PD, LK) screened the remaining articles and decided independently if all eligibility criteria were met. Occurring disagreements were resolved by a third reviewer (HB).

### Data extraction

Data was extracted by two reviewers (PD, CK) independently. Again, occurring disagreements were resolved by a third reviewer (HB).

### Risk of bias

Quality of the included studies was assessed by two independent reviewers (PD, LK) with the Cochrane risk of bias tool 2.0 provided by the Cochrane Collaboration [30]. According to this version of the risk of bias tool the studies have to be rated on five risk domains for potential biases to arise from (1) the randomization process, (2) deviations from intended interventions, (3) missing outcome data, (4) measurement of the outcome, and (5) selection of the reported results. By the help of this tool each domain and therefore each study was rated and lead to judgments of either “low risk of bias”, “some concern”, or “high risk of bias”.

### Data analysis

Random effects meta-analyses were conducted for the chosen efficacy outcome measures (Table 1) in comparison to two control group clusters: passive control group comprising wait list control groups (WLC), treatment as usual and no treatment and active control groups with or without face to face (f2f) treatment. Effect sizes for continuous outcomes were reported as hedge´s g with 95% confidence intervals.

**Table 1:** Study Characteristics I

Authors	Pub. year	Focus	Sample size IG/CG	% female	Mean age	Age range	Eligibility criteria	Control group(s)	Primary outcome	Time point of post-treatment measurement in weeks	Country
Conaughton et al.	2017	Anx	21/21	14.3	14.3	8–12	Diagnosis of Asperger´s Syndrome made by a health professional and diagnosis of SAD, SP, SEP or GAD with CSR ≥ 4 according to ADIS-C/P	WLC	CSR (ADIS-C/P) for Anx	14	Australia
Ip et al.	2016	Dep	130/127	68.1	14.63	13–17	CES-D score 12–40	AC (website)	CES-D	17	China (Hong Kong)
Jolstedt et al.	2018	Anx	66/65	53	9.95	8–12	Primary diagnosis of SAD, GAD, SP, SEP or PD according to ADIS-C/P with CSR ≥ 4	AC (directed play)	CSR (ADIS-C/P) for Anx	13	Sweden
Lindqvist et al.	2020	Dep	38/38	80	16.6	15–18	QIDS-SR score ≥ 10 and meet criteria for unipolar MDD according to M.I.N.I. 7.0	AC (supportive contact)	QIDS-SR	10	Sweden
March et al.	2009	Anx	34/29	54.8	9.45	7–12	Primary diagnosis of Anx (except OCD, PD or PTBS) according to ADIS-C/P with CSR ≥ 4	WLC	CSR (ADIS-C/P) for Anx	10	Australia
Moeini et al.	2019	Dep	64/64	100	16.7	15–18	CES-D score of 10–45	TAU	CES-D	16	Iran
Nordh et al.	2021	Anx	51/52	77	14.1	10–17	Principal diagnosis of SAD according to ADIS-C/P with CSR ≥ 4	AC (supportive contact and symptom monitoring)	CSR (ADIS-C/P) for Anx	10	Sweden
Rickhi et al.	2015	Dep	18/13	84	15.3	12–18	DSM-IV-TR criteria for major depressive disorder (mild to moderate severity) and obtained a CDRS-R score of 40–70 or HAMD score of 12–24	WLC	CDRS-R	8	Canada
Schniering et al.	2022	Anx and Dep	45/46	66	14.29	12–17	Diagnosis of Dep and Anx according to ADIS-C/P with CSR ≥ 4	WLC	SCAS-Y (Anx) SMFQ-Y (Dep)	8	Australia
Spence et al.	2011	Anx	44/44/27	41	13.98	12–18	Diagnosis of SAD, SEP, GAD or SP according to ADIS-C/P with CSR ≥ 4	CBTf2f and WLC	CSR (ADIS-C/P) for Anx	12	Australia
Spence et al.	2017	Anx	47/48/30	60	11.28	8–17	Diagnosis of SAD according to ADIS-C/P with CSR ≥ 4	iCBT and WLC	CSR (ADIS-C/P) for Anx	12	Australia
Stjerneklar et al.	2019	Anx	35/35	79	15.03	13–17	primary diagnosis of Anx according to ADIS-C/P with CSR ≥ 4	WLC	CSR (ADIS-C/P) for Anx	14	Denmark
Tilfors et al.	2013	Anx	10/9	89	16.5	15–21	Diagnosis of SAD according to SCID F module	WLC	SPSQ-C	9	Sweden
Topooco et al.	2018	Dep	34/37	96	17.04	15–19	At least 5 symptoms or diagnosis of MDD according to M.I.N.I.	AC (supportive contact and symptom monitoring)	BDI-II	8	Sweden
Topooco et al.	2019	Dep	35/35	96	17.5	15–19	At least 5 symptoms or diagnosis of MDD according to M.I.N.I.	AC (supportive contact and symptom monitoring)	BDI-II	8	Sweden
Vigerland et al.	2016	Anx	46/47	51	10.1	8–12	Primary diagnosis of GAD, PD, SAD, SEP or SP according to ADIS-C/P with CSR ≥ 4	WLC	CSR (ADIS-C/P) for Anx	10	Sweden
Waite et al.	2019	Anx	30/30	65	14.7	13–18	Primary diagnosis of GAD, PD, SAD, SEP or SP according to ADIS-C/P with CSR ≥ 4	WLC	CSR (ADIS-C/P) for Anx	10	UK

*Abbreviations:* AC = active control, ADIS-C/P = Anxiety Disorders Interview Schedule for Children and Parents, Anx = anxiety disorder, BDI-II = Beck-Depressions-Inventory II, CBT = cognitive behavior therapy, CES-D = Center for Epidemiological Studies Depression, CDRS-R = Children’s Depression Rating Scale - Revised, CG = control group, CSR = clinician severity rating, Dep = depression, f2f = face to face, GAD = generalized anxiety disorder, HAMD = Hamilton Rating Scale for Depression, iCBT = internet-based cognitive behavior therapy, IG = intervention group, M.I.N.I. = Mini International Neuropsychiatric Interview, MDD = major depressive disorder, OCD = obsessive compulsive disorder, PD = panic disorder, PTBS = post-traumatic stress disorder, QIDS-SR = Quick Inventory of Depressive Symptomatology Self- Report, SAD = social anxiety disorder, SCAS = Spence’s Children’s Anxiety Scale, SCID = Structured Clinical Interview for DSM-IV, SEP = separation anxiety disorder, SMFQ-Y = Short Mood and Feelings Questionnaire - Youth, SP = specific phobia, SPSQ-C = Social Phobia Screening Questionnaire for Children and adolescents, TAU = treatment as usual, WLC = wait-list control

Note: Due to the small amount of included trials the planned separation into four separate control groups clusters was abandoned and two clusters were formed instead, passive control groups (i.e. WLC, TAU or no treatment) and active control groups (i.e. active control with f2f treatment and active control without f2f) as well a combination of both (i.e. active and passive control groups).

Statistical heterogeneity was evaluated using the Q statistic, further quantified using the I^2^ statistic as well as visualized via forest plots. A common rule of thumb is 25% low-, 50% moderate- and 75% high-statistical heterogeneity [31]. To further account for statistical heterogeneity a random effects meta-analysis model was used in the analyses. A potential publication bias will be visually examined via funnel plots.

Potential subgroup effects were investigated for different variables. The following *moderators*: (1) symptom severity pre-intervention (low vs. moderate vs. severe), (2) age (children (13 years and younger) vs. adolescents (≥ 13 to 18 years) vs. mixed age samples), (3) male and female sample compositions (0 to ≤ 40% male = high female sample, ≤ 40% female = high male sample, > 40 to < 60% male and female = balanced sample), (4) clinically relevant symptom level vs. diagnosed disorders (elevated vs. diagnosed), *mediators*: (5) human support during the IMIs (guided vs. unguided), *study design variables* (6) outcome type (self-report vs. observer rated) and 9) measurement timepoints (post randomization 0–6 months vs. post-randomization 6–12 months vs. post-randomization > 12 months), (7) publication year and (8) RoB rating (low vs. some concern vs. high) and were inspected. If subgroup analyses were not feasible (< 3 studies per subgroup), moderators were reported qualitatively.

### Negative effects

Negative effects were evaluated descriptively according to the definitions of Rozental and colleagues [22], differentiating between deterioration (worsening of the target symptoms, monitored by validated outcome measure), adverse events (negative effects probably emerging from the treatment and perceived as adverse, causing worsening of target symptoms, not monitored by validated outcome measures), severe adverse events (negative effects that occur during treatment, that require some form of high intensity treatment response), novel symptoms (new psychological symptoms, unrelated to target symptoms, may or may not be associated to treatment), dropout (number of participants prematurely ending treatment), non-response (lack of predicted positive effect on target symptoms) and unwanted events (all other negative effects that occur during the treatment, may or may not be related to treatment, does not necessarily influence treatment outcome).

## Results

### Study selection

**Fig. 1:**
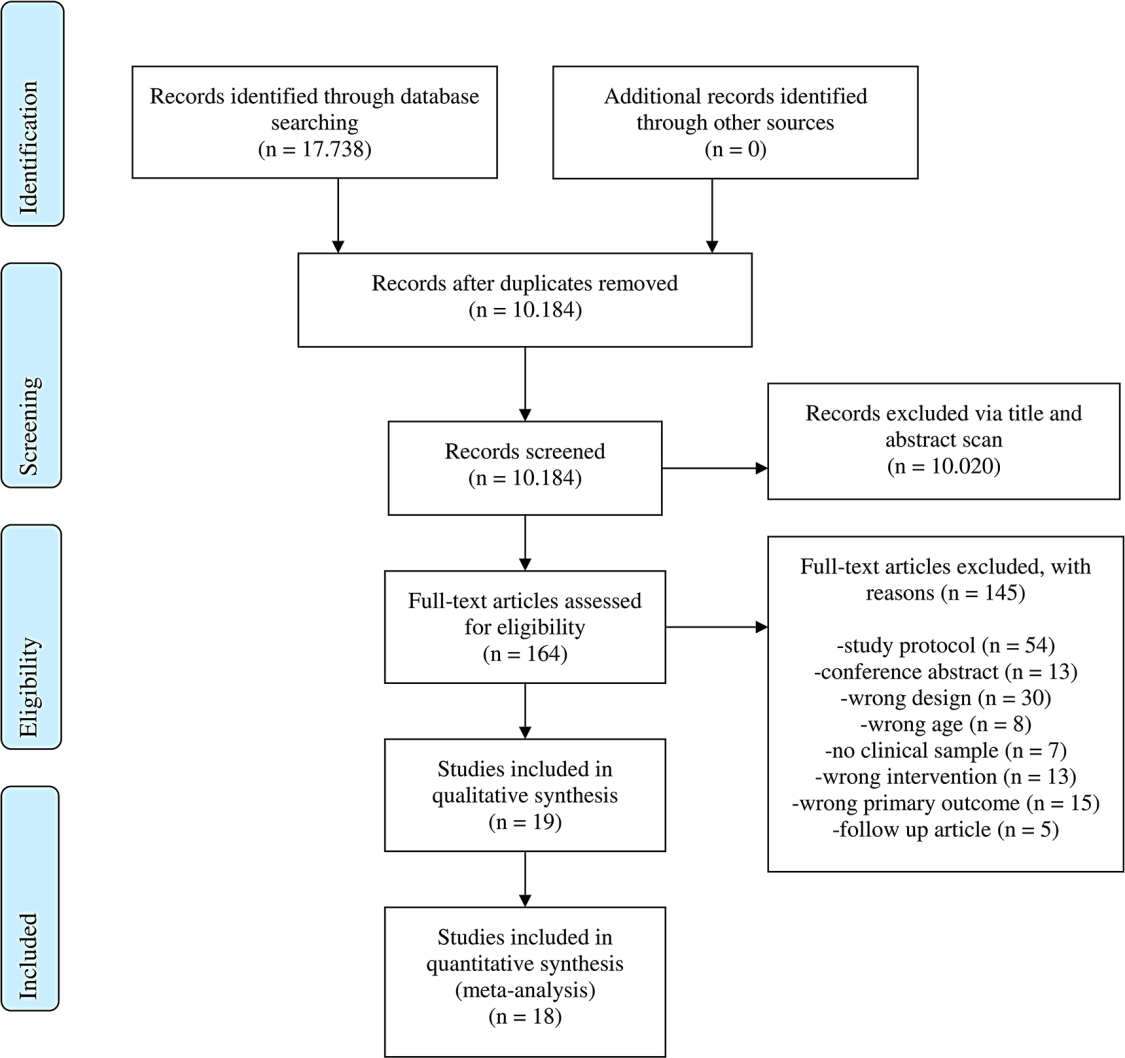
PRISMA Flow Chart

A total of 17,738 articles were initially identified and after the removal of duplicates 10,184 remained for further screening. At the end 17 individual studies with 17 trials fulfilled all inclusion criteria (Fig. 1). One cluster randomized study (cRCT; [32]) was in accordance with the Cochrane guidelines [33] not included into the statistical analysis but reported qualitatively.

### Study characteristics

The studies included in the present review are 16 RCTs and one cRCT. Tables 1 and 2 show all main study characteristics of the included studies and implemented IMIs. 10 studies focused on anxiety disorders [34–39, 17, 40–43], six on depression [44–48, 32] and one on depression and anxiety disorders [42]. 88.2% of RCTs were conducted in western countries, in total 1,465 participants were randomized and the sample sizes were ranging from 19 to 257 participants, with a mean size of *n* = 85.88 (SD = 54.73), the total mean age was 14.05 years (SD = 2.56). Most studies were either balanced between sexes (k = 4) or had a higher proportion of female participants (k = 12), only one study had a higher proportion of male participants. Used control group designs were various forms of attention control without f2f treatment (k = 6), attention control with f2f treatment (k = 1), TAU (k = 1) and WLC (k = 9). Most studies focused on adolescents (k = 8) or had mixed samples (k = 6), only three exclusively on children. All except two studies used a pre-existing or an interview-based diagnosis as an inclusion criterion, the other two studies used elevated self-report symptom scores. The vast majority of studies implemented IMIs based on CBT (k = 15), one study used internet-based psychodynamic therapy (IPDT) as theoretical foundation and another study used a spirituality-based IMI. The post-treatment assessment was on average 11.42 weeks (SD = 2.91) after the initial baseline assessments. All except two studies used some form of human guidance during the IMI. The two other studies only provided technical support or provided only automated support presented in videos during the intervention tasks.

**Table 2:** Study Characteristics II

Authors	Pub. year	Recruitment	Intervention background	Guidance (yes or no)	How guided	Nr. of modules	Duration in weeks	Average completed modules; mean (SD)
Conaughton et al.	2017	Recruited through referral from general practitioners, mental health professionals, school guidance officers, teachers, parents and through media publicity	iCBT	yes	Weekly messages with therapist and one short phone call midway through the program	10 and 2 booster sessions	10	6.71 (2.99)
Ip et al.	2016	Recruited at three secondary schools	iCBT	no	Monthly reminders by phone call or by messages through email and social media, technical support	10	35	Median = 3 (interquartile range = 5)
Jolstedt et al.	2018	Recruited through newspaper advertisement; and through referral from clinical research unit at child and adolescent mental health service or primary care centers	iCBT	yes	Weekly asynchronous support from clinician	12	12	7.91 (3.38)
Lindqvist et al.	2020	Recruited through social media, information via schools, youth centers and youth mental health care providers	IPDT	yes	Weekly 30-minute chat sessions, additional support on demand	8	8	5.8 (2.4)
March et al.	2009	Recruited through media releases and information packages send to schools; through referral from parents, teachers, guidance officers, other mental health professionals	iCBT	yes	Weekly responses from online therapist to homework and session activities, automated e-mails before and after each session, 2 telephone therapist contact	10 and 2 booster sessions	10	7.5 (3.1)
Moeini et al.	2019	Recruited at all-girls schools	iCBT	yes	Constant online assistance from a psychiatrist via message, text message reminders	8	12	na
Nordh et al.	2021	Recruited through advertisement at the child and adolescent mental health services clinics in Stockholm and newspapers	iCBT	yes	3 times 20-30-minute video call sessions with a therapist, asynchronous support	10	10	7.53 (2.6)
Schniering et al.	2022	Recruited at the Centre for Emotional Health at Macquarie University	iCBT	yes	8 times 30-minute telephone sessions with a therapist (caregiver participated in 4)	8	8	na
Spence et al.	2011	Recruited through advertisements in school newsletters, newspaper articles, television and radio interviews, and through referral from school guidance officers, general practitioners, and other mental health professionals	iCBT	yes	E-mail feedback following each session, 15-min telephone call following session 5, personalized automated e-mails after each session and as a reminder	10 and 2 booster sessions	10	7.5
Spence et al.	2017	Recruited across Australia via schools, parent groups, mental health professionals, guidance officers, the media and facebook	iCBT	yes	E-mail feedback following each session, 15-min telephone call following session 5, personalized automated e-mails after each session and as a reminder	10 and 2 booster sessions	10	children: 4.75 (na), teenager: 4 (na)
Stjerneklar et al.	2019	Recruited through postings on the website or recommendations from local community health services	iCBT	yes	Weekly phone calls (20-min) with feedback and assistance	8	14	5.4 (2.37)
Topooco et al.	2018	Recruited through social media, schools and youth mental health organisations	iCBT	yes	Weekly synchronous chat sessions	8	8	6.48 (2.43)
Topooco et al.	2019	Recruited through social media posts, schools, youth centers and clinics	iCBT	yes	Weekly synchronous chat sessions	8	8	6.2 (2.28)
Vigerland et al.	2016	Recruited through media advertisements	iCBT	yes	Online contact through written messages and written feedback on worksheets, at least 3 phone calls	11	10	9.7 (1.8)
Waite et al.	2019	Recruited through primary and secondary care services	iCBT	yes	Weekly individualized written feedback, telephone call following session 5, personalized automated e-mails after each session and as a reminder	10 and 2 booster sessions	10	na
Rickhi et al.	2015	Recruited through mails, presentations, local media, social media, local educational institutions, health professionals, social workers, and community organizations that provide services to youth	LEAP	no	Only content is presented by a professional host who introduces and guides participants through the program materials	8	8	IG: 72% (*n* = 13) full completion, 11% (*n* = 2) more than half completion, 17% (*n* = 3) less than half completion CG: 92% (*n* = 12) full completion, 8% (*n* = 1) less than half completion
Tilfors et al.	2013	Recruited through regional newspaper articles, school staff and advertisements in high schools	iCBT	yes	Weekly written feedback, e-mail reminders	9	9	2.9 (na)

*Abbreviations*: CG = control group, iCBT = internet-based cognitive behavior therapy, IG = intervention group, IPDT = Internet-based psychodynamic therapy, LEAP = Life Enrichment and Appreciation Program, na = not available, SD = standard deviation

### Meta-analyses

If studies evaluating an IMI targeting either anxiety or depression included additional outcomes for the respective other disorder, only the outcome measuring the symptoms of the target disorder was used in the statistical analysis. This procedure was chosen to assure that the outcomes included in the analyses were assessed in samples with clinically relevant symptoms of the mental disorder under study.

Two studies used more than two comparison groups. The first one [37] had one IMI intervention group, one f2f CBT intervention group and one WLC group. To be able to include this study in the analysis and in accordance with the Cochran Handbook for Systematic Reviews [33] only the comparison between the IMI and f2f CBT group was included. The second study [38] had one IMI group with an intervention specialized for social anxiety disorders, one IMI group with an intervention for anxiety disorders in general and one WLC group. In accordance with the Cochran Handbook for Systematic Reviews [33] the two IMI groups were combined and compared to the WLC group.

#### Efficacy anxiety

**Fig. 2:**
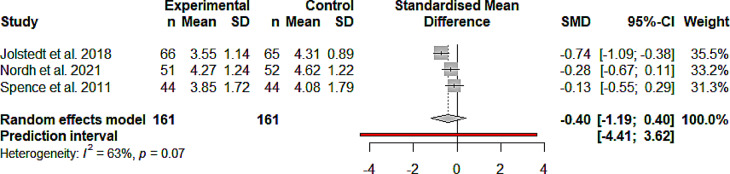
Forest plot of anxiety IMIs compared to Active control groups

IMIs focusing on anxiety disorders showed no significant improvement at post-treatment compared to active control groups (g = -0.4; CI -1.19 to 0.4; k = 3; *n* = 322; *p* = 0.16; Fig. 2). Heterogeneity was not indicated by the Q value (Q_2_ = 5.43; *p* = 0.066). I^2^ = 63.1% indicates moderate heterogeneity.

**Fig. 3:**
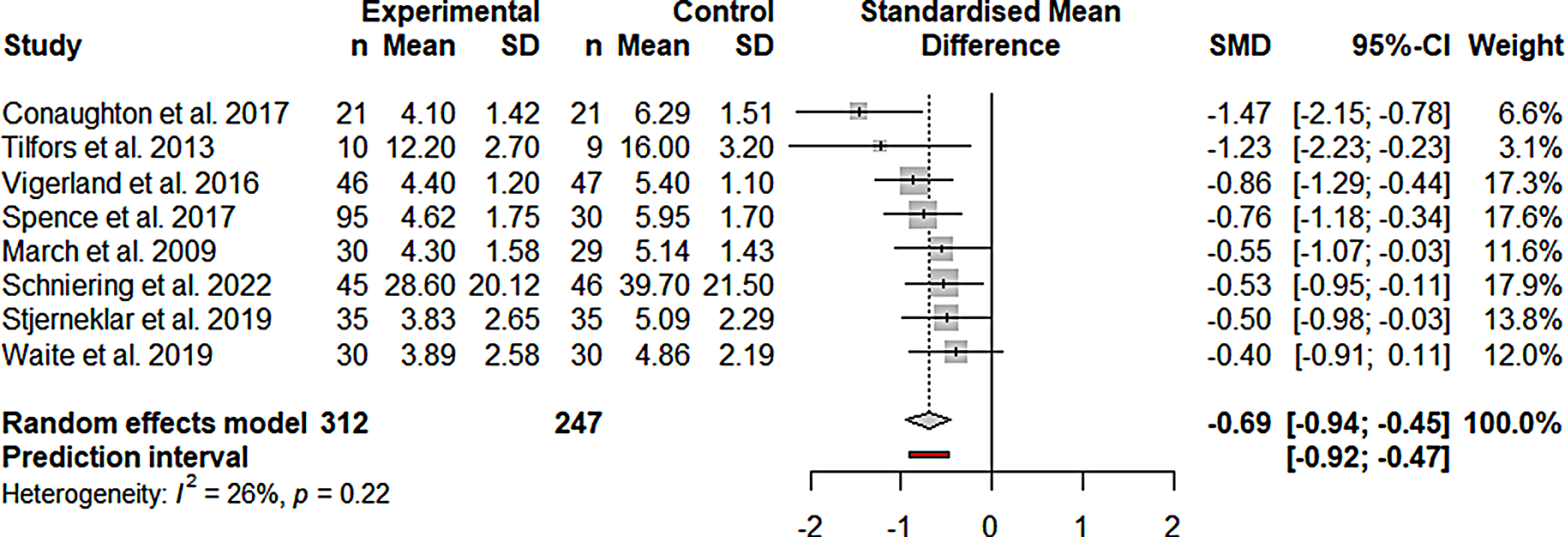
Forest plot of anxiety IMIs compared to passive control groups

IMIs focusing on anxiety disorders showed a significant improvement at post-treatment if compared to passive control groups (g = -0.69; CI -0.94 to -0.45; k = 8; *n* = 559; *p* ≤ 0.001; Fig. 3). Heterogeneity was not indicated by the Q value (Q_7_ = 9.42; *p* = 0.22). I^2^ = 25.7% indicates low heterogeneity.

#### Efficacy depression

**Fig. 4:**
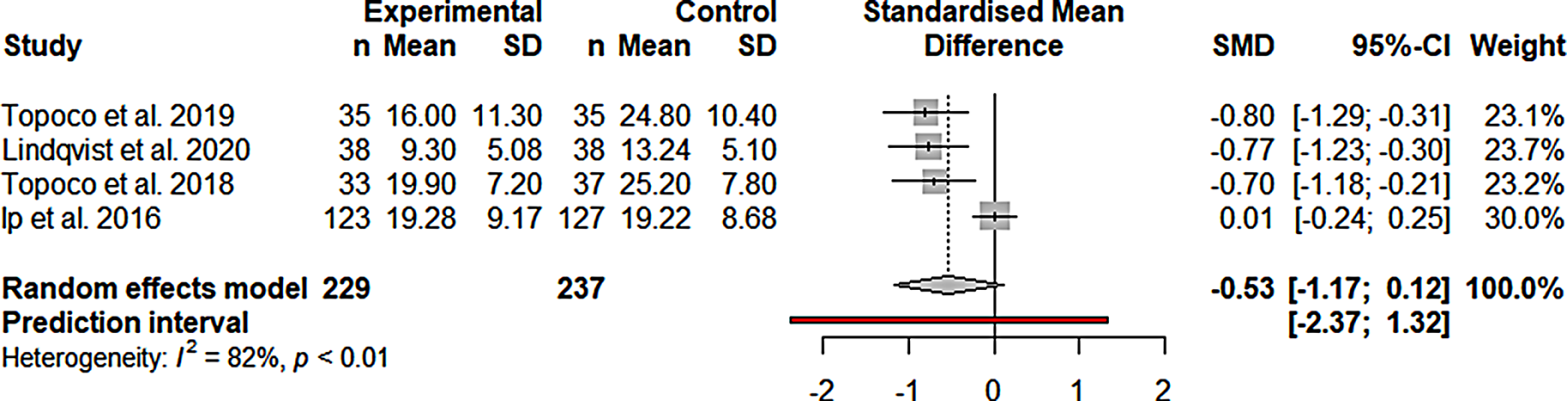
Forest plot of depression IMIs compared to active control groups

For depression outcomes no significant improvement at post-treatment compared to active control groups could be observed (g = -0.53; CI -1.17 to 0.12; k = 4; *n* = 466; *p* = 0.08; Fig. 4). Heterogeneity was indicated by a significant Q value (Q_3_ = 16.23; *p* = 0.001). I^2^ = 81.5% indicates substantial heterogeneity.

**Fig. 5:**
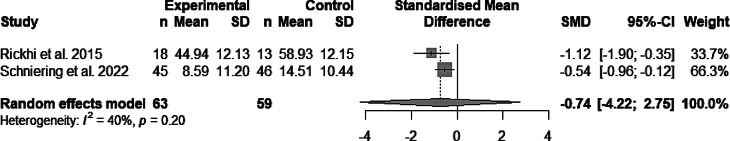
Forest plot of depression IMIs compared to passive control groups

For depression outcomes no significant improvement at post-treatment if compared to passive control groups was shown (g = -0.74; CI -4.22 to 2.75; k = 2; *n* = 122; *p* = 0.23; Fig. 5). Heterogeneity was not indicated by the Q value (Q_5_ = 1.67; *p* = 0.2). I^2^ = 40.2% indicates moderate heterogeneity.

### Subgroup analyses

#### Subgroups anxiety

Subgroup analyses for anxiety outcomes in comparison to active control groups were not carried out due to the small number of trials per subgroup.

Subgroup analyses for anxiety outcomes in comparison to passive control groups were carried out for symptom severity pre-intervention (Q_1_ = 6,97; *p* = 0.0083), indicating higher efficacy for moderate symptom levels (g = -0.85; CI -1.23 to -0.48; k = 5) compared with low symptom levels (g = -0.49; CI -0.64 to -0.32; k = 3). All other pre-defined subgroup analyses were not carried out due to the small number of trials per subgroup.

#### Subgroups depression

Subgroup analyses for depression outcomes in comparison to active or passive control groups separate were not carried out due to the small number of trials per subgroup.

### Negative effects across all included studies

All included studies reported numbers that allowed for conclusions about drop-out rates at the post-assessment, only a few studies reported drop-out rates directly. Furthermore, all but four studies reported numbers that showed the number of participants that reliably improved or no longer met diagnostic criteria, allowing for conclusions about the number of participants that did not improve. Apart from theses information, only six studies (35.29%) reported additional details about negative effects [35, 36, 39, 45–47]. Deterioration rates and all other questionnaires were only reported as summaries without quantitative values that could be used for meta-analytic analyses, therefore, the findings on negative effects are reported qualitatively (Tables 3 and 4).

**Table 3:** Negative Effects of included studies. Overview: How were they measured?

Study	Open/Closed Questions	Validated Negative Effect Questionnaire
Conaughton et al. 2017	na	na
Ip et al. 2016	na	na
Jolstedt et al. 2018	Unclear if open or closed questions: Self-reported adverse events. Had to rate if impact was at the time of the event or still at post-treatment. Measured at post-treatment in IG and CG.	na
Lindqvist et al. 2020	Open question: Assess any potential negative effects. Measured pot-treatment in IG. Closed questions: QIDS-A17-SR. Measured at post-treatment in IG and CG.	na
March et al. 2009	na	na
Moeini et al. 2019	na	na
Nordh et al. 2021	Unclear if open or closed questions: “During treatment, youths and parents were continuously asked to report any adverse events.”	NEQ (symptoms subscale) at post-treatment in IG and CG.
Rickhi et al. 2015	na	na
Schniering et al. 2022	na	na
Spence et al. 2011	na	na
Spence et al. 2017	na	na
Stjerneklar et al. 2019	Open question: If closed question true, additional qualitative information was asked. Measured at post-treatment only in IG. Closed question: Whether the treatment had caused them/their child to feel worse (“Not true”, “true” or “partly true”, 3-item scale).	na
Tillfors et al. 2011	na	na
Topooco et al. 2018	Open question: Report negative treatment-related experiences. Measured at post-treatment in IG. Closed questions: BDI-II. Measured at post-treatment in IG and CG. Significant deterioration was defined as deteriorating 30% or more on the BDI-II score, baseline to post-treatment. Additionally, the PHQ-9 was implemented on a weekly basis to monitor for depression severity in both groups.	na
Topooco et al. 2019	Closed questions: BDI-II. Measured at post-treatment in IG and CG. Significant deterioration was defined as deteriorating 30% or more on the BDI-II score, baseline to post-treatment. Additionally, the short version of the MFQ-13 and the suicidal ideation item from the PHQ-9 were implemented on a weekly basis to monitor depression severity on both groups.	na
Vigerland et al. 2016	na	na
Waite et al. 2019	na	na

*Abbreviations:* BDI-II = Beck-Depressions-Inventory II, CG = control group, IG = intervention group, MFQ-13 = Mood and Feelings Questionnaire-13, na = not available, NEQ = Negative Effects Questionnaire, PHQ-9 = Patient Health Questionnaire-9, QIDS-SR = Quick Inventory of Depressive Symptomatology Self- Report

#### Drop-out rates

Drop-out rates before the post-treatment assessment were derivable from all included studies, ranging from 2.2 to 25.3% with a mean across all studies of 11.7% (SD = 7.2) in the IG and from 0 to 30% with a mean of 7.1% (SD = 7.7) in the CG. Separated by index disorder of the intervention we calculated a drop-out rate of 11.52% (SD = 7.93) for anxiety and 12.05% (SD = 6.54) for depression. It was, however, mostly unclear if the reported drop-out rates were study, assessment or intervention drop-outs (Table 4 for more details).

**Table 4:** Negative effects of included studies. Overview: What was found?

Study	Focus	Adverse events	Novel symptoms	Unwanted events	Drop-out^1^ before post-treatment assessment % (n) IG/ % (n) CG	Response or non-response post-treatment	Deterioration	Severe adverse events
Conaughton et al. 2017	Anx	na	na	na	14.3 (3)/14.3 (3)	19% (*n* = 4) IG, 0% (*n* = 0) CG of ITT sample no longer met diagnostic criteria	na	na
Ip et al. 2016	Dep	na	na	na	5.4 (7)/0 (0)	na	na	na
Jolstedt et al. 2018	Anx	At least one self-reported negative event: IG 25.8% (*n* = 17) and CG 24.6% (*n* = 16), no significant difference (*p* = 0.786). Depressive symptoms (IG = 3.7% and CG 3.6%), anxiety symptoms (IG = 16.7% and CG = 23.6%), anger/tantrums (IG = 5.6% and CG = 1.8%) and somatic symptoms (IG = 5.6% and CG = 0%).	na	na	9.1 (6)/6.2 (4)	48% (*n* = 29) IG, 15% (*n* = 9) CG of completers no longer met diagnostic criteria	na	No severe adverse events were found in either condition.
Lindqvist et al. 2020	Dep	At least one self-reported negative event in the IG 18% (*n* = 6). Feelings of loneliness (3%), increased awareness of feelings of anger and that this was painful and distressing in the short term (3%), feelings of distress in connection with facing previously avoided thoughts and feelings (6%).	na	Found the treatment format stressful (6%), feelings of shame in connection with not completing exercises on time (3%).	13.2 (5)/26 (1)	56% (*n* = 19) IG / 21% (*n* = 8) CG of ITT sample showed reliable improvement on the QIDS-SR^2^	0% in the IG and 8.11% (*n* = 3) in the CG deteriorated reliably on the QIDS-SR. No clear definition of a reliable deterioration.	No serious adverse events were reported during the trial.
March et al. 2009	Anx	na	na	na	25 (10)/12.1 (4)	30% (*n* = 9) IG, 10.3% (*n* = 3) CG of completers no longer met diagnostic criteria	na	na
Moeini et al. 2019	Dep	na	na	na	25 (16)/6.3 (4)	na	na	na
Nordh et al. 2021	Anx	At least one self-reported negative effect: IG 39% (*n* = 20) and CG 29% (*n* = 15), all of them reported disturbed sleep or increased anxiety, increased conflicts with parents 10% in the IG (*n* = 5) and 4% (*n* = 2) and suicidal ideation in the CG 8% (*n* = 4) in the IG and 12% (*n* = 6). Comparisons between the groups were all none-significant (*p* > 0.05).	na	na	3.9 (2)/0 (0)	na	na	One suicide attempt was reported and managed in the CG.
Rickhi et al. 2015	Dep		na	na	5.5 (1)/0 (0)	na	na	na
Schniering et al. 2022	Anx and Dep	na	na	na	11.1 (5)/10.9 (5)	43.8% (*n* = 20) IG, 20.9% (*n* = 10) CG no longer met diagnostic criteria of both disorders	na	na
Spence et al. 2011	Anx	na	na	na	6.8 (3)/9.1 (4)	34.1% (*n* = 15) iCBT, 29.5% (*n* = 13) CBT, 3.7% (*n* = 1) WLC of ITT sample no longer met diagnostic criteria	na	na
Spence et al. 2017	Anx	na	na	na	25.3 (24)/10 (3)	14.7% (*n* = 7) iCBT, 3.3% (*n* = 1) WLC of ITT sample no longer met diagnostic criteria	na	na
Stjerneklar et al. 2019	Anx	3% (*n* = 1) rated the statement “Whether the treatment had caused them/their child to feel worse” to be true, while 10% (*n* = 3) rated it to be ‘partly true’. None of the additional information indicated further clinical interventions.	na	na	8.6 (3)/ 11.4 (4)	69% (*n* = 22) IG, 26% (*n* = 8) CG of ITT sample showed reliable improvement on SCAS-C^2^ 40% (*n* = 14) IG, 16% (*n* = 5) CG of ITT sample no longer met diagnostic criteria	na	na
Tillfors et al. 2011	Anx		na	na	10 (1)/0 (0)	60% (*n* = 9) IG of completers showed reliable improvement on SPSQ-C^2^	na	na
Topooco et al. 2018	Dep	At least one self-reported negative effect: IG 15% (*n* = 5) either “at times feeling worse while processing treatment content”,	na	or “occasional stress due to tempo and workload in treatment.”	11.8 (4)/2.7 (1)	IG 60.6% (*n* = 20), CG 32.4% (*n* = 12) showed ≥ 30% decrease, IG 42.4% (*n* = 14), CG 13.5% (*n* = 5) showed ≥ 50% decrease on BDI-II (ITT sample)	For completers, 3% (*n* = 1) in the IG and 8% (*n* = 3) in the CG deteriorated significantly (increase of ≥ 30% on the BDI-II from baseline to post-treatment). With missing cases categorized as having deteriorated significantly, the rate in the IG changed to 12.1% (*n* = 4).	Not reported, except that no participant had to be excluded from the study due to deterioration.
Topooco et al. 2019	Dep		na	na	11.4 (4)/0(0)	46% (*n* = 16) IG, 11% (*n* = 4) CG of ITT sample showed reliable improvement on BDI-II^2^ 56% (*n* = 15) IG, 27% (*n* = 7) CG of ITT sample no longer met diagnostic criteria	0% in the IG and KG deteriorated significantly (increase of ≥ 30% on the BDI-II from baseline to posttreatment). Missing cases categorized as having deteriorated significantly, the rate in the IG changed to 11% (*n* = 4).	Not reported, except that one participant (IG) deteriorated significantly during treatment, directed to standard care services while staying in the study.
Vigerland et al. 2016	Anx		na	na	2.2 (1)/4.2 (2)	20% (*n* = 9) IG, 7% (*n* = 3) CG of ITT sample no longer met diagnostic criteria	na	na
Waite et al. 2019	Anx		na	na	10 (3)/30 (9)	40% (*n* = 12) IG, 23.3% (*n* = 7) CG of ITT sample no longer met diagnostic criteria	na	na

*Notes:*^1^ = Average amount of completed modules can be found in Table 3. ^2^ = RCI according to Jacobson et al. 1991. *Abbreviations*: BDI-II = Beck-Depressions-Inventar II, CBT = cognitive behavior therapy, CG = control group, IG = intervention group, iCBT = internet-based cognitive behavior therapy, ITT = intention to treat, na = not available, QIDS-SR = Quick Inventory of Depressive Symptomatology Self- Report, RCI = Reliable Change Index, SCAS = Spence’s Children’s Anxiety Scale, SPSQ-C = Social Phobia Screening Questionnaire for Children and adolescents, WLC = wait-list cont

#### (Non-)Response or Remission

Response to the treatment was reported in 13 studies. 10 studies [41, 35, 34, 42, 37–39, 46, 47, 17, 40] reported the number of participants that no longer met diagnostic criteria after the intervention, ranging from 13.7 to 56% with a mean of 34.5% (SD = 13.7) in the IG and from 0 to 27% with a mean of 12.7% (SD = 9.2) in the CG. This translates to an average of non-remission, according to the definition of still meeting diagnostic criteria after the intervention, of 65.5% in the IG and 87,3% in the CG.

Five studies [39, 43, 45–47] reported the number of participants that reliably improved on the outcome measure, defined as improving by 30% or according to the reliable change index (RCI) [49]. In the four studies reporting outcomes according to the RCI, participants improved on the outcome measure in the IG on average 34.5% (SD = 12.7) ranging from 46 to 69% and in CG on average 12.7% (SD = 9.2) ranging from 11 to 26%. The other study showed that 60.6% in the IG and 32.4% in the CG showed a decrease of ≥ 30% on the outcome measure. This again translates to an average of non-response after the treatment of 39.4–65.6% in the IG compared to 67.6–87.3% in the CG.

#### Deterioration rates

Deterioration rates were reported in three (15.8%) depression studies [45–47]. One study [45] reported reliable deterioration rates on the QIDS-SR post-treatment of 0% in the intervention group (IG) and 8.1% (*n* = 3) in the control group (CG). Another study reported that 3% (*n* = 1) of the completers in the IG and 8% (*n* = 3) in the CG deteriorated significantly on the BDI-II score post-treatment (defined as increase of ≥ 30% on the BDI-II from baseline to post-treatment), while the number rose to 12.1% (*n* = 4) in the IG if missing cases were categorized as having deteriorated significantly as well [46]. The third study reported that 0% of the completers in the IG or CG had deteriorated significantly (again defined as increase of ≥ 30% on the BDI-II), if counting missing cases as having deteriorated significantly 11% (*n* = 4) in the IG and 0% (*n* = 0) in the CG reached this definition [47].

#### Adverse events, novel symptoms and unwanted events assessed through open questions

In three studies (21.05%) open questions were used to assess negative effects [39, 45, 46], in two studies (10.5%) it was unclear if open or closed questions were used [35, 36]. One study [35] reported depression symptoms (IG = 3.7% and CG 3.6%), anger/tantrums (IG = 5.6% and CG = 1.8%) and somatic symptoms (IG = 5.6% and CG = 0%), with no significant difference between IG 25.8% (*n* = 17) and CG 24.6% (*n* = 16) (*p* = 0.786). Most negative effects had an impact at the time of the event (IG = 9.1% and CG = 16.9%), less at post-treatment (IG = 1.5% and CG = 9.2%). In a second study [45] 18% (*n* = 6) reported at least one negative effect of the following for the IG: feelings of loneliness (3%), increased awareness of feelings of anger and that this was painful and distressing in the short term (3%), feelings of distress in connection with facing previously avoided thoughts and feelings (6%) and found the treatment format stressful (6%), feelings of shame in connection with not completing exercises on time (3%). Sterneklar and colleagues [39] reported that one participant (3%) rated the statement, “Whether the treatment had caused them/their child to feel worse”, to be true, while 10% (*n* = 3) rated it to be partly true. None of the additional information collected in the open question indicated the need for any further clinical interventions according to the authors [39]. Finally, Topooco et al. [46] reported that 15% (*n* = 5) in the IG indicated negative effects such as occasional stress due to the pace and workload in the treatment, or at times feeling worse while processing treatment content.

#### Adverse events, novel symptoms and unwanted events assessed through validated negative effects questionnaires

Only one study [36] used a validated questionnaire which was developed especially for the assessment of negative effects during psychotherapeutic treatments, namely the symptom subscale of the negative effects questionnaire [50]. The authors reported that 39% (*n* = 20) in the IG and 29% (*n* = 15) in the CG reported at least some form of negative effects in relation to the treatment. All of them reported sleep disturbances or increased anxiety, 10% in the IG (*n* = 5) and 4% (*n* = 2) in the CG reported increased conflicts with parents additionally, 8% (*n* = 4) in the IG and 12% (*n* = 6) reported suicidal ideation. None of the reported negative effects were significantly different between the two groups [36].

#### Serious adverse events

Serious adverse events were mentioned in three studies [35, 36, 45]. In two of these studies no serious adverse events were found [35, 45], while Nordh and colleagues [36] reported one suicide attempt in the CG. Two further studies reported that participants who experience deterioration did not have to be excluded due to the experienced deterioration [46] and that one participant (IG) that showed significant deterioration was directed to the standard care services but was not excluded from the study [47].

### Risk of bias assessment

**Fig. 6:**
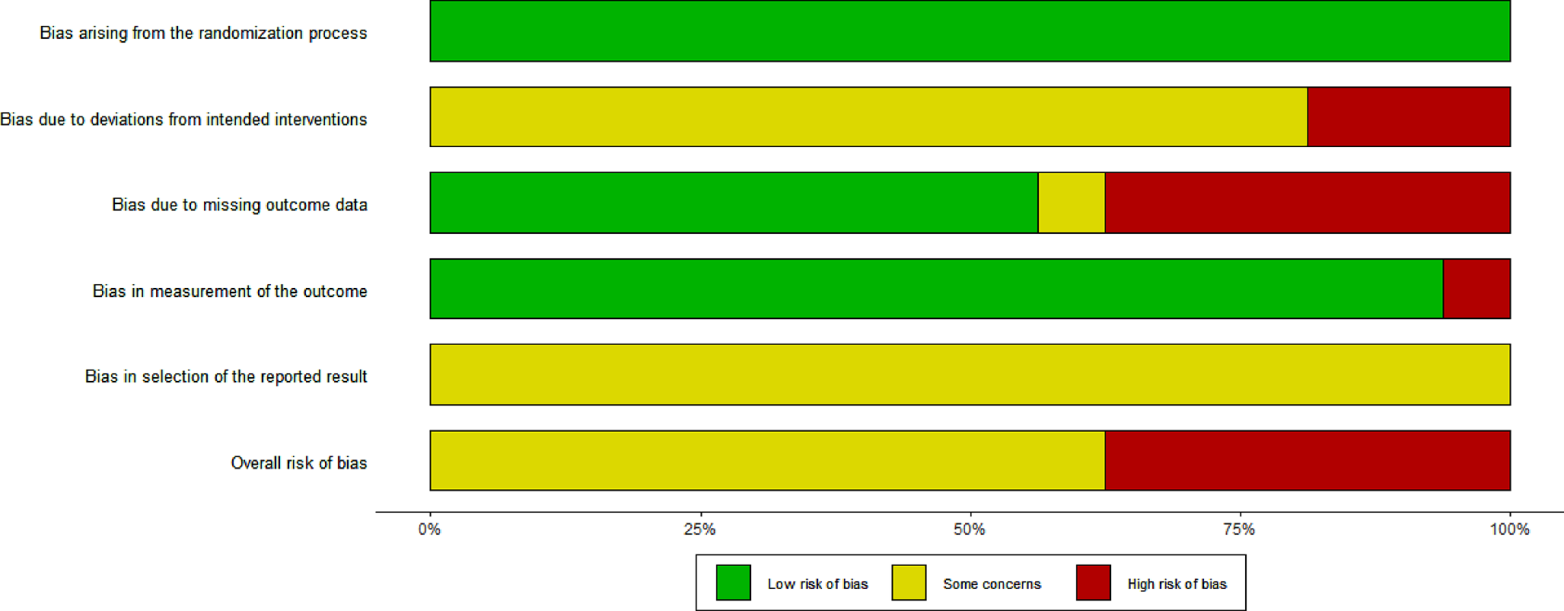
Risk of bias assessment plot - all trials

The risk of bias assessment for all included studies is illustrated in Fig. 6. Inter-rater reliability between the two raters was acceptable (Cohen´s Kappa = 0.69).

### Assessment of publication bias

**Fig. 7:**
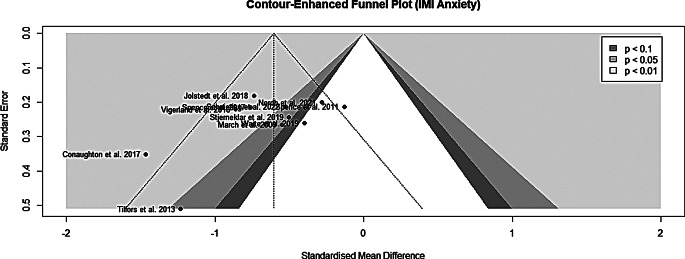
Funnel plot for anxiety IMI trials

**Fig. 8:**
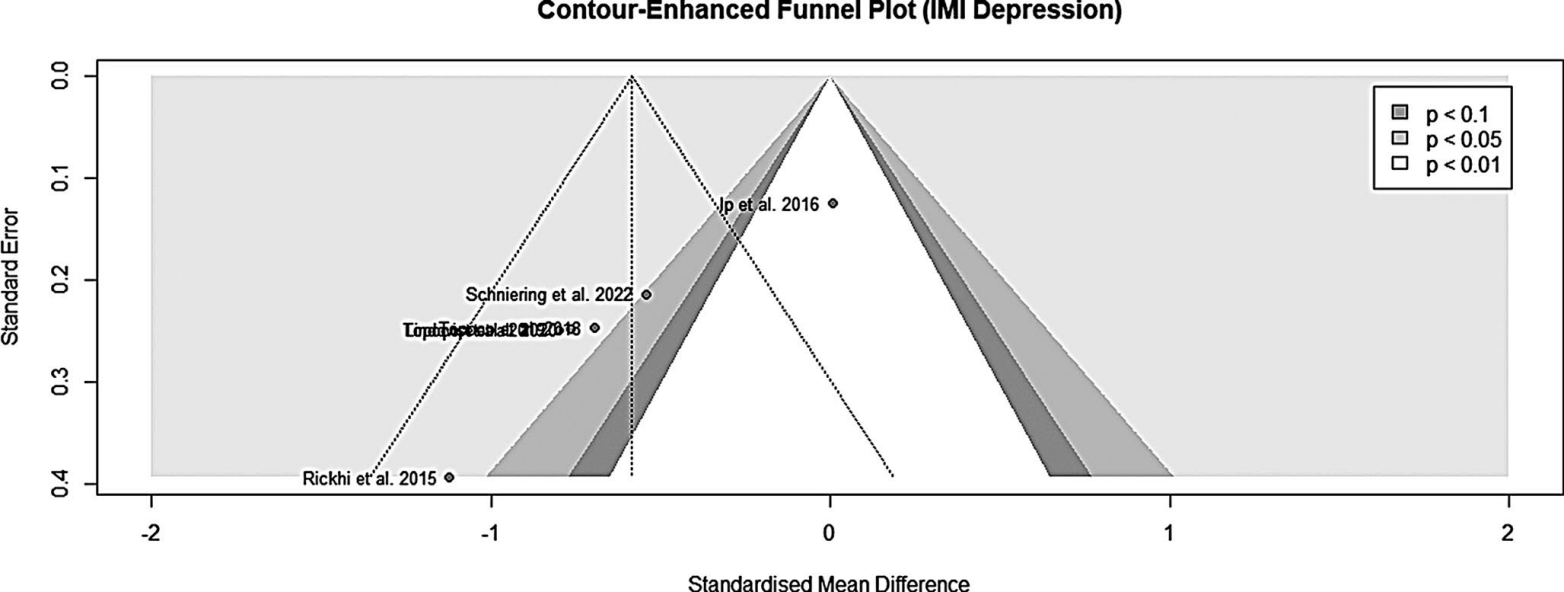
Funnel plot for depression IMI trials

Publication bias was investigated by the means of funnel plots for studies targeting anxiety disorder (Fig. 7) and depression (Fig. 8). The funnel plots exhibited no clear indication of publications bias; however, the small number of studies should be considered. Due to the insufficient number of studies investigating IMIs targeting depression, Egger´s regression test [51] was only performed for IMIs aimed at anxiety disorders (t_9_ = -1.59; *p* = 0.147). Also, quantitively no clear indication of funnel plot asymmetry and therefore publication bias could be found for studies investigating IMIs targeting anxiety disorders.

## Discussion

The present systematic review and meta-analysis was conducted to evaluate and summarize the current evidence base available for internet- and mobile based interventions targeting anxiety disorders or depression in children and adolescents with clinically relevant symptoms, thereby updating prior meta-analytical evidence (12, 27, 13). Through a comprehensive search via four databases 10,184 unique articles have been identified and 17 studies were included in the qualitative review with a total of 1,720 participants and 16 studies in the quantitative meta-analytical analyses with a total of 1,593 participants. Results showed a significant moderate effect size for IMIs targeting anxiety disorders compared to passive control groups, similar to previous work [12, 13, 27]. However, the findings indicate neither a significant benefit of IMIs targeting anxiety compared to active control groups nor for IMIs targeting depression.

The moderate efficacy of IMIs targeting anxiety disorders shown in the present review should be viewed in light of the limited number of available trials. Integrating the present findings into the literature, we confirmed a moderate effect in comparison to passive control groups also for samples up to 18 years [27]. The inclusion criteria of the present review of a sample age limit at 18 years did not show a difference in effect size compared to reviews including samples ranging from 12 to 25 years [12]. This could indicate, that IMIs might be similar in efficacy for all young individuals up to the age of 25 years. However, this should only be said with some certainty for adolescents and young adults. The present review and previous work [27] has still not found a conclusive answer to the question of differential efficacy for children. Although previous work indicates a positive moderating effect of higher age [11, 52], a clear comparison between children and adolescents was still not possible. This brings us to a general lack of enough studies with children and adolescents’ samples. It is therefore difficult to meaningfully extract the necessary information on a level that differentiates enough. This conundrum was prevalent in the present review during the forming of the control group clusters. The initially planned four separate control group clusters could not be formed, hence, we binarily differentiated only between passive control groups and active control groups which might has leveled out control group specific effects to some extent.

For the evaluation of IMIs targeting depression six studies were included. Neither the comparison against active control groups nor against passive control groups indicated a significant benefit of depression IMIs. As previously, this null-finding has to be viewed in light of the limited number of available trials and the observed heterogeneity. Previous reviews that included more trials, either due to a broader inclusion of intervention types [27], age groups [12] or the combination of control groups [13], indicated a positive effect of depression IMIs on depression outcomes. However, considering the limitations of including young adults, various symptom levels and combining control groups [12, 13] we have to conclude that the evidence is still inconclusive and on more differentiated levels of analyses often just not available. A finding that was also advocated by Moshe and colleagues [16] in a recent meta-analyses that included different age groups.

Regarding the pre-planned subgroup analyses only one comparison was feasible, IMIs targeting anxiety disorders compared to passive control groups and baseline symptom severity. Here the significant result indicates a positive association between higher symptom severity and intervention efficacy. This differential effect was already reported for adult samples [53, 54], however, in contrast one review with children and adolescents reported reduced efficacy in diagnosed populations compared to samples with a mixed group of diagnosed and undiagnosed individuals up to the age of 25 years [52]. With regard to the pre-planned analyses, our review highlights the need to further evaluate the differential roles of moderating and mediating factors in IMIs for children and adolescents. Available reviews with adults samples indicate the importance of scrutinizing different moderators and mediators to further our understanding of for whom and how IMIs are most effective [55–57].

Increased awareness of the importance of examining aspects beyond effectiveness is also a major finding of our review regarding negative effects. All trials allowed for some conclusions regarding negative effects, however, most trials can be regarded as having covered this topic insufficiently. Reported post-assessment drop-out rates ranged from 0 to 30%, mirroring size and span of drop-outs in adult f2f psychotherapy [58], f2f psychotherapy for children and adolescents [59, 60] and IMIs for adults [61]. Intervention non-response or non-remission, showed to be in the range of 40–65%, which is likely higher than adult f2f psychotherapy [62–64] or f2f psychotherapy for children and adolescents [65, 66]. Only six studies [36, 37, 40, 46–48] mentioned additional negative effects that did or did not occur during their studies. Of these remaining six studies, all used some form of self-designed open and/or closed questions. Three studies [45–47] reported deterioration rates in the range of 5–10% on validates questionnaires. Similar rates were found in IMI research with adults samples [24–26] or for f2f treatments [67]. Information about serious adverse events were only reported in three studies [35, 36, 45]. All reported cases of SAEs were considered to be unrelated to the treatment evaluated in the studies.

One reason for the regular shortcomings of negative effects assessments might be found in a quote of Daniel Kahneman “The brains of humans contain a mechanism that is designed to give priority to bad news.” [68]. Hence, it seems partly understandable to feel the urge to omit these kinds of information to not taint the promising results of studies. However, the evaluation of a treatment will never be complete if one does not consider its potential or actual negative effects. Therefore, possibly our brains and ourselves might become better at integrating bad news in form of negative effects, if bad news were more commonly reported in the research literature. If they would not be reported so scarcely they might just be seen as another piece of information in the evaluation process without prioritizing them ahead of others, as has been advocated before [22]. This leaves the question of how negative effects could and should be reported as well as integrated in the decision process of which intervention to implement. Researchers have argued that a combination of quantitative deterioration rates and qualitative self-reports should be used [22]. During sorting and systematizing the reported information about negative effects from the included trials it became apparent that comparability in measurements seems to be a factor that allows for meaningful conclusions between trials but certainly risks to neglect the individuality of the whole topic. Therefore building on Rozental and colleagues [22] future RCTs should first of all start to include reports of negative effects on a regular basis by using a combination of quantitative (e.g. deterioration rates, (S)AEs) and qualitative information (e.g. open questions, negative effect questionnaires; semi-structured interviews with patients that indicated negative effects quantitatively). Secondly, a common language of what constitutes as negative effect and how many distinct categories can or should be differentiated before the categories start to blur, as one might note about the presently used categories by Rozental and colleagues [22]. Such a common language is particularly important if the comparability should reach across the limits of the own research field.

To complete the picture limitations of the present review have to be mentioned as well. One major limitation is the limited amount of eligible trials. In general, it seems necessary to conduct more RCTs on IMIs for children and adolescents as target population to allow for meaningful conclusion on a fine-grained level of specificity. This leads to the second limitation, the combination of different control groups into only two control group clusters, which might have leveled out control group specific effects. Such an approach was only chosen due to the limited amount of included trials. The third limitation concerns the reported drop-out rates. It was mostly not possible to differentiate between study, intervention or assessment drop-out. However, details on intervention drop-outs are especially important when evaluating potential treatment offers.

## Conclusion

Taken together, the results of the present review indicate a moderate benefit of IMIs targeting anxiety disorders in participants up to 18 years with clinically relevant symptoms against passive control groups. Results for IMIs targeting depression are inconclusive. Beyond these general statements for children and adolescents the evidence regarding a more differentiated conclusion on who (and who not) benefits from which IMI under what circumstances best is largely lacking [55–57]. The reporting of negative effects, furthermore, clearly highlights another important lack of evidence. It seems mandatory that the research field examining IMIs for children and adolescent moves forward and beyond the current too often expressed hope of what works for adults might surely work for all and surely again be of no harm to children and adolescents. From our perspective hope should not be our guide when it comes to urgently needed scalable, evidence-based mental health care for children and adolescents, but a far more comprehensive evidence-base on the efficacy and possible negative effects, as well as moderating and mediating factors of these intervention outcomes.

## Electronic supplementary material

Below is the link to the electronic supplementary material.

Supplementary Material 1

## Abbreviations

ADIS-C/P: Anxiety Disorders Interview Schedule for Children and Parents
BDI-II: Beck-Depressions-Inventar II
CBT: cognitive behavior therapy
CDRS-R: Children’s Depression Rating Scale – Revised
CENTRAL: Cochrane controlled trial register
CES-D: Center for Epidemiological Studies Depression
CG: control group
CI: confidence interval
cRCT: cluster randomized controlled trial
CSR: clinician severity rating
EMBASE: Excerpta Medica Database
e.g.: exempli gratia
f2f: face to face
GAD: generalized anxiety disorder
HAMD: Hamilton Rating Scale for Depression
iCBT: internet-based cognitive behavior therapy
IG: intervention group
IMIs: internet- and mobile-based interventions
IPDT: Internet-based psychodynamic therapy
LEAP: Life Enrichment and Appreciation Program
MDD: major depressive disorder
M.I.N.I.: Mini International Neuropsychiatric Interview
OSF: Open Science Framework
Ovid: Ovid Technologies
PD: panic disorder
PHQ-9: Patient Health Questionnaire-9
PRISMA: Preferred Reporting Items for Systematic Reviews and Meta-Analyses
PTBS: post-traumatic stress disorder
QIDS-SR: Quick Inventory of Depressive Symptomatology Self- Report
RCI: Reliable Change Index
RCT: randomized controlled trial
SAD: social anxiety disorder
SAD: serious adverse event
SCAS: Spence’s Children’s Anxiety Scale
SCID: Structured Clinical Interview for DSM-IV
SD: standard deviation
SEP: separation anxiety disorder
SMFQ-Y: Short Mood and Feelings Questionnaire – Youth
SP: specific phobia
SPSQ-C: Social Phobia Screening Questionnaire for Children and adolescents
TAU: treatment as usual
WLC: wait-list control group
