# Supplementary material for: Internet- and mobile-based anxiety and depression interventions for children and adolescents: efficacy and negative effects - a systematic review and meta-analysis
Source: Eur Child Adolesc Psychiatry. 2024 Mar 2;34(1):101–21. doi: 10.1007/s00787-024-02404-y (PMC11805827; doi:10.1007/s00787-024-02404-y)
Supplement: Supplementary file 1 — Supplementary Material 1 [file 787_2024_2404_MOESM1_ESM.docx]

Appendix 1. Search string

| Search # | MEDLINE |  | CENTRAL |  | PSYCHINFO |  | EMBASE |
| --- | --- | --- | --- | --- | --- | --- | --- |
|  | e-Health |  |  |  |  |  |  |
| 1 | exp internet/ | 1 | exp internet/ | 1 | exp internet/ | 1 | exp internet/ |
| 2 | exp mobile phone/ | 2 | exp mobile phone/ | 2 | exp mobile phone/ | 2 | exp mobile phone/ |
| 3 | exp mobile applications/ | 3 | exp mobile applications/ | 3 | exp mobile applications/ | 3 | exp mobile applications/ |
| 4 | internet*.ab,ti. | 4 | internet*.ab,ti. | 4 | internet*.ab,ti. | 4 | internet*.ab,ti. |
| 5 | online*.ab,ti. | 5 | online*.ab,ti. | 5 | online*.ab,ti. | 5 | online*.ab,ti. |
| 6 | web*.ab,ti. | 6 | web*.ab,ti. | 6 | web*.ab,ti. | 6 | web*.ab,ti. |
| 7 | computer*.ab,ti. | 7 | computer*.ab,ti. | 7 | computer*.ab,ti. | 7 | computer*.ab,ti. |
| 8 | mobile*.ab,ti. | 8 | mobile*.ab,ti. | 8 | mobile*.ab,ti. | 8 | mobile*.ab,ti. |
| 9 | electronic*.ab,ti. | 9 | electronic*.ab,ti. | 9 | electronic*.ab,ti. | 9 | electronic*.ab,ti. |
| 10 | phone*.ab,ti. | 10 | phone*.ab,ti. | 10 | phone*.ab,ti. | 10 | phone*.ab,ti. |
| 11 | app.ab,ti. | 11 | mobile app*.ab,ti. | 11 | mobile app*.ab,ti. | 11 | mobile app*.ab,ti. |
| 12 | application.ab,ti. | 12 | software app*.ab,ti. | 12 | software app*.ab,ti. | 12 | software app*.ab,ti. |
| 13 | tele*.ab,ti. | 13 | tele*.ab,ti. | 13 | tele*.ab,ti. | 13 | tele*.ab,ti. |
| 14 | cyber*.ab,ti. | 14 | cyber*.ab,ti. | 14 | cyber*.ab,ti. | 14 | cyber*.ab,ti. |
| 15 | digital*.ab,ti. | 15 | digital*.ab,ti. | 15 | digital*.ab,ti. | 15 | digital*.ab,ti. |
| 16 | smartphone*.ab,ti. | 16 | smartphone*.ab,ti. | 16 | smartphone*.ab,ti. | 16 | smartphone*.ab,ti. |
| 17 | virtual*.ab,ti. | 17 | virtual*.ab,ti. | 17 | virtual*.ab,ti. | 17 | virtual*.ab,ti. |
| 118 | 1-17 OR | 119 | 1-17 OR | 120 | 1-17 OR | 119 | 1-17 OR |
|  | Intervention Psychotherapy |  |  |  |  |  |  |
| 18 | exp psychotherapy/ | 18 | exp psychotherapy/ | 18 | exp psychotherapy/ | 18 | exp psychotherapy/ |
| 19 | psychotherap*.ab,ti. | 19 | psychotherap*.ab,ti. | 19 | psychotherap*.ab,ti. | 19 | psychotherap*.ab,ti. |
| 20 | self-help.ab,ti. | 20 | self-help.ab,ti. | 20 | self-help.ab,ti. | 20 | self-help.ab,ti. |
| 21 | selfhelp.ab,ti. | 21 | selfhelp.ab,ti. | 21 | selfhelp.ab,ti. | 21 | selfhelp.ab,ti. |
| 22 | intervention*.ab,ti. | 22 | intervention*.ab,ti. | 22 | intervention*.ab,ti. | 22 | intervention*.ab,ti. |
| 23 | treatment*.ab,ti. | 23 | treatment*.ab,ti. | 23 | treatment*.ab,ti. | 23 | treatment*.ab,ti. |
| 119 | 18-23 OR | 120 | 18-23 OR | 121 | 18-23 OR | 120 | 18-23 OR |
| 125 | 118 AND 119 | 126 | 119 AND 120 | 127 | 120 AND 121 | 126 | 119 AND 120 |
|  | Online Psychotherapy |  |  |  |  |  |  |
| 24 | exp telemedicine/ | 24 | exp telemedicine/ | 24 | exp telemedicine/ | 24 | exp telemedicine/ |
| 25 | exp computer-assisted therapy/ | 25 | exp computer-assisted therapy/ | 25 | exp computer-assisted therapy/ | 25 | exp computer assisted therapy/ |
| 26 | exp distance counseling/ | 26 | exp distance counseling/ | 26 | / | 26 | exp distance counseling/ |
|  |  |  |  | 118 | exp online therapy/ |  |  |
| 27 | exp e-counseling/ | 27 | exp e-counseling/ | 27 | / | 27 | exp e-counseling/ |
| 28 | exp teletherapy/ | 28 | exp teletherapy/ | 28 | exp teletherapy/ | 28 | exp teletherapy/ |
| 29 | distance counseling.ab,ti. | 29 | distance counseling.ab,ti. | 29 | distance counseling.ab,ti. | 29 | distance counseling.ab,ti. |
| 30 | e-health*.ab,ti. | 30 | e-health*.ab,ti. | 30 | e-health*.ab,ti. | 30 | e-health*.ab,ti. |
| 31 | ehealth*.ab,ti. | 31 | ehealth*.ab,ti. | 31 | ehealth*.ab,ti. | 31 | ehealth*.ab,ti. |
| 32 | m-health*.ab,ti. | 32 | m-health*.ab,ti. | 32 | m-health*.ab,ti. | 32 | m-health*.ab,ti. |
| 33 | mhealth*.ab,ti. | 33 | mhealth*.ab,ti. | 33 | mhealth*.ab,ti. | 33 | mhealth*.ab,ti. |
| 34 | e-treat*.ab,ti. | 34 | e-treat*.ab,ti. | 34 | e-treat*.ab,ti. | 34 | e-treat*.ab,ti. |
| 35 | etreat*.ab,ti. | 35 | etreat*.ab,ti. | 35 | etreat*.ab,ti. | 35 | etreat*.ab,ti. |
| 36 | telemedicine*.ab,ti. | 36 | telemedicine*.ab,ti. | 36 | telemedicine*.ab,ti. | 36 | telemedicine*.ab,ti. |
| 37 | tele-medicine*.ab,ti. | 37 | tele-medicine*.ab,ti. | 37 | tele-medicine*.ab,ti. | 37 | tele-medicine*.ab,ti. |
| 38 | telehealth*.ab,ti. | 38 | telehealth*.ab,ti. | 38 | telehealth*.ab,ti. | 38 | telehealth*.ab,ti. |
| 39 | tele-health*.ab,ti. | 39 | tele-health*.ab,ti. | 39 | tele-health*.ab,ti. | 39 | tele-health*.ab,ti. |
| 40 | telebased*.ab,ti. | 40 | telebased*.ab,ti. | 40 | telebased*.ab,ti. | 40 | telebased*.ab,ti. |
| 41 | tele-based*.ab,ti. | 41 | tele-based*.ab,ti. | 41 | tele-based*.ab,ti. | 41 | tele-based*.ab,ti. |
| 42 | internetbased.ab,ti. | 42 | internetbased.ab,ti. | 42 | internetbased.ab,ti. | 42 | internetbased.ab,ti. |
| 43 | internet-based.ab,ti. | 43 | internet-based.ab,ti. | 43 | internet-based.ab,ti. | 43 | internet-based.ab,ti. |
| 44 | internetdelivered.ab,ti. | 44 | internetdelivered.ab,ti. | 44 | internetdelivered.ab,ti. | 44 | internetdelivered.ab,ti. |
| 45 | internet-delivered.ab,ti. | 45 | internet-delivered.ab,ti. | 45 | internet-delivered.ab,ti. | 45 | internet-delivered.ab,ti. |
| 46 | etherap*.ab,ti. | 46 | etherap*.ab,ti. | 46 | etherap*.ab,ti. | 46 | etherap*.ab,ti. |
| 47 | e-therap*.ab,ti. | 47 | e-therap*.ab,ti. | 47 | e-therap*.ab,ti. | 47 | e-therap*.ab,ti. |
| 48 | emental health*.ab,ti. | 48 | emental health*.ab,ti. | 48 | emental health*.ab,ti. | 48 | emental health*.ab,ti. |
| 49 | e-mental health*.ab,ti. | 49 | e-mental health*.ab,ti. | 49 | e-mental health*.ab,ti. | 49 | e-mental health*.ab,ti. |
| 50 | telecare*.ab,ti. | 50 | telecare*.ab,ti. | 50 | telecare*.ab,ti. | 50 | telecare*.ab,ti. |
| 51 | tele-care*.ab,ti. | 51 | tele-care*.ab,ti. | 51 | tele-care*.ab,ti. | 51 | tele-care*.ab,ti. |
| 52 | icbt*.ab,ti. | 52 | icbt*.ab,ti. | 52 | icbt*.ab,ti. | 52 | icbt*.ab,ti. |
| 53 | i-cbt*.ab,ti. | 53 | i-cbt*.ab,ti. | 53 | i-cbt*.ab,ti. | 53 | i-cbt*.ab,ti. |
| 54 | ccbt*.ab,ti. | 54 | ccbt*.ab,ti. | 54 | ccbt*.ab,ti. | 54 | ccbt*.ab,ti. |
| 55 | c-cbt*.ab,ti. | 55 | c-cbt*.ab,ti. | 55 | c-cbt*.ab,ti. | 55 | c-cbt*.ab,ti. |
| 56 | internetintervention*.ab,ti. | 56 | internetintervention*.ab,ti. | 56 | internetintervention*.ab,ti. | 56 | internetintervention*.ab,ti. |
| 57 | internet-intervention*.ab,ti. | 57 | internet-intervention*.ab,ti. | 57 | internet-intervention*.ab,ti. | 57 | internet-intervention*.ab,ti. |
| 120 | 24-57 OR | 121 | 24-57 OR | 122 | 24-57 AND 118 OR | 121 | 24-57 OR |
| 126 | 125 OR 120 | 127 | 126 OR 121 | 128 | 127 OR 122 | 127 | 126 OR 121 |
|  | Depression |  |  |  |  |  |  |
| 58 | exp mood disorder/ | 58 | exp mood disorder/ | 58 | exp mood disorder/ | 58 | exp mood disorder/ |
| 59 | exp depression/ | 59 | exp depression/ | 59 | exp depression/ | 59 | exp depression/ |
| 60 | exp depressive disorder/ | 60 | exp depressive disorder/ | 60 | / | 60 | exp depressive disorder/ |
| 61 | depressi*.ab,ti. | 61 | depressi*.ab,ti. | 61 | depressi*.ab,ti. | 61 | depressi*.ab,ti. |
| 62 | mood disorder*.ab,ti. | 62 | mood disorder*.ab,ti. | 62 | mood disorder*.ab,ti. | 62 | mood disorder*.ab,ti. |
| 63 | affective disorder*.ab,ti. | 63 | affective disorder*.ab,ti. | 63 | affective disorder*.ab,ti. | 63 | affective disorder*.ab,ti. |
| 64 | dysthymi*.ab,ti. | 64 | dysthymi*.ab,ti. | 64 | dysthymi*.ab,ti. | 64 | dysthymi*.ab,ti. |
| 65 | major depression.ab,ti. | 65 | major depression.ab,ti. | 65 | major depression.ab,ti. | 65 | major depression.ab,ti. |
| 66 | seasonal affective disorder.ab,ti. | 66 | seasonal affective disorder.ab,ti. | 66 | seasonal affective disorder.ab,ti. | 66 | seasonal affective disorder.ab,ti. |
| 67 | bipolar*.ab,ti. | 67 | bipolar*.ab,ti. | 67 | bipolar*.ab,ti. | 67 | bipolar*.ab,ti. |
| 121 | 58-67 OR | 122 | 58-67 OR | 123 | 58-67 OR | 122 | 58-67 OR |
|  | Anxiety |  |  |  |  |  |  |
| 68 | exp anxiety disorder/ | 68 | exp anxiety disorder/ | 68 | exp anxiety disorder/ | 68 | exp anxiety disorder/ |
| 69 | exp phobia/ | 69 | exp phobia/ | 69 | exp phobia/ | 69 | exp phobia/ |
| 70 | exp panic/ | 70 | exp panic/ | 70 | exp panic/ | 70 | exp panic/ |
| 71 | exp test anxiety/ | 71 | exp test anxiety/ | 71 | exp test anxiety/ | 71 | exp test anxiety/ |
| 72 | exp separation anxiety/ | 72 | exp separation anxiety/ | 72 | exp separation anxiety/ | 72 | exp separation anxiety/ |
| 73 | exp generalized anxiety disorder/ | 73 | exp generalized anxiety disorder/ | 73 | exp generalized anxiety disorder/ | 73 | exp generalized anxiety disorder/ |
|  |  |  |  | 119 | exp anxiety neurosis/ | 118 | exp anxiety neurosis/ |
| 74 | exp anxiety/ | 74 | exp anxiety/ | 74 | exp anxiety/ | 74 | exp anxiety/ |
| 75 | exp social anxiety disorder/ | 75 | exp social anxiety disorder/ | 75 | exp social anxiety disorder/ | 75 | exp social anxiety disorder/ |
| 76 | exp agoraphobia/ | 76 | exp agoraphobia/ | 76 | exp agoraphobia/ | 76 | exp agoraphobia/ |
| 77 | anxi*.ab,ti. | 77 | anxi*.ab,ti. | 77 | anxi*.ab,ti. | 77 | anxi*.ab,ti. |
| 78 | panic*.ab,ti. | 78 | panic*.ab,ti. | 78 | panic*.ab,ti. | 78 | panic*.ab,ti. |
| 79 | phobi*.ab,ti. | 79 | phobi*.ab,ti. | 79 | phobi*.ab,ti. | 79 | phobi*.ab,ti. |
| 80 | agoraphobi*.ab,ti. | 80 | agoraphobi*.ab,ti. | 80 | agoraphobi*.ab,ti. | 80 | agoraphobi*.ab,ti. |
| 81 | social anxiety disorder*.ab,ti. | 81 | social anxiety disorder*.ab,ti. | 81 | social anxiety disorder*.ab,ti. | 81 | social anxiety disorder*.ab,ti. |
| 82 | generalized anxiety disorder*.ab,ti. | 82 | generalized anxiety disorder*.ab,ti. | 82 | generalized anxiety disorder*.ab,ti. | 82 | generalized anxiety disorder*.ab,ti. |
| 83 | generalised anxiety disorder*.ab,ti. | 83 | generalised anxiety disorder*.ab,ti. | 83 | generalised anxiety disorder*.ab,ti. | 83 | generalised anxiety disorder*.ab,ti. |
| 84 | OCD.ab,ti. | 84 | OCD.ab,ti. | 84 | OCD.ab,ti. | 84 | OCD.ab,ti. |
| 85 | obsessive compulsive disorder*.ab,ti. | 85 | obsessive compulsive disorder*.ab,ti. | 85 | obsessive compulsive disorder*.ab,ti. | 85 | obsessive compulsive disorder*.ab,ti. |
| 86 | social anxi*.ab,ti. | 86 | social anxi*.ab,ti. | 86 | social anxi*.ab,ti. | 86 | social anxi*.ab,ti. |
| 87 | seperation anxi*.ab,ti. | 87 | seperation anxi*.ab,ti. | 87 | seperation anxi*.ab,ti. | 87 | seperation anxi*.ab,ti. |
| 88 | hoarding.ab,ti. | 88 | hoarding.ab,ti. | 88 | hoarding.ab,ti. | 88 | hoarding.ab,ti. |
| 122 | 68-88 OR | 123 | 68-88 OR | 124 | 68-88 AND 119 OR | 123 | 68-88 AND 118 OR |
| 127 | 121 OR 122 | 128 | 122 OR 123 | 129 | 123 OR 124 | 128 | 122 OR 123 |
|  | Studyn Design |  |  |  |  |  |  |
|  |  | 118 | exp randomized controlled trials as topic/ |  |  |  |  |
| 89 | exp randomized controlled trial/ | 89 | exp randomized controlled trial/ | 89 | exp randomized controlled trial/ | 89 | exp randomized controlled trial/ |
| 90 | randomi?ed.ab,ti. | 90 | randomi?ed.ab,ti. | 90 | randomi?ed.ab,ti. | 90 | randomi?ed.ab,ti. |
| 91 | placebo.ab,ti. | 91 | placebo.ab,ti. | 91 | placebo.ab,ti. | 91 | placebo.ab,ti. |
| 92 | randomly.ab,ti. | 92 | randomly.ab,ti. | 92 | randomly.ab,ti. | 92 | randomly.ab,ti. |
| 93 | trial.ab,ti. | 93 | trial.ab,ti. | 93 | trial.ab,ti. | 93 | trial.ab,ti. |
| 94 | groups.ab,ti. | 94 | groups.ab,ti. | 94 | groups.ab,ti. | 94 | groups.ab,ti. |
| 95 | controlled clinical trial.ab,ti. | 95 | controlled clinical trial.ab,ti. | 95 | controlled clinical trial.ab,ti. | 95 | controlled clinical trial.ab,ti. |
| 96 | random*.ab,ti. | 96 | random*.ab,ti. | 96 | random*.ab,ti. | 96 | random*.ab,ti. |
| 97 | randomi?ed controlled trial.ab,ti. | 97 | randomi?ed controlled trial.ab,ti. | 97 | randomi?ed controlled trial.ab,ti. | 97 | randomi?ed controlled trial.ab,ti. |
| 98 | controll*.ab,ti. | 98 | controll*.ab,ti. | 98 | controll*.ab,ti. | 98 | controll*.ab,ti. |
| 99 | rct.ab,ti. | 99 | rct.ab,ti. | 99 | rct.ab,ti. | 99 | rct.ab,ti. |
| 123 | 89-99 OR | 124 | 89-99 + 118 OR | 125 | 89-99 OR | 124 | 89-99 OR |
|  | Population |  |  |  |  |  |  |
| 100 | exp adolescent/ | 100 | exp adolescent/ | 100 | / | 100 | exp adolescent/ |
| 101 | exp child/ | 101 | exp child/ | 101 | / | 101 | exp child/ |
| 102 | exp teenager/ | 102 | exp teenager/ | 102 | / | 102 | exp teenager/ |
| 103 | child*.ab,ti. | 103 | child*.ab,ti. | 103 | child*.ab,ti. | 103 | child*.ab,ti. |
| 104 | childhood.ab,ti. | 104 | childhood.ab,ti. | 104 | childhood.ab,ti. | 104 | childhood.ab,ti. |
| 105 | teen*.ab,ti. | 105 | teen*.ab,ti. | 105 | teen*.ab,ti. | 105 | teen*.ab,ti. |
| 106 | adolescen*.ab,ti. | 106 | adolescen*.ab,ti. | 106 | adolescen*.ab,ti. | 106 | adolescen*.ab,ti. |
| 107 | young pe*.ab,ti. | 107 | young pe*.ab,ti. | 107 | young pe*.ab,ti. | 107 | young pe*.ab,ti. |
| 108 | youth*.ab,ti. | 108 | youth*.ab,ti. | 108 | youth*.ab,ti. | 108 | youth*.ab,ti. |
| 109 | boy*.ab,ti. | 109 | boy*.ab,ti. | 109 | boy*.ab,ti. | 109 | boy*.ab,ti. |
| 110 | girl*.ab,ti. | 110 | girl*.ab,ti. | 110 | girl*.ab,ti. | 110 | girl*.ab,ti. |
| 111 | young adult*.ab,ti. | 111 | young adult*.ab,ti. | 111 | young adult*.ab,ti. | 111 | young adult*.ab,ti. |
| 112 | juvenile*.ab,ti. | 112 | juvenile*.ab,ti. | 112 | juvenile*.ab,ti. | 112 | juvenile*.ab,ti. |
| 113 | youngster*.ab,ti. | 113 | youngster*.ab,ti. | 113 | youngster*.ab,ti. | 113 | youngster*.ab,ti. |
| 114 | student*.ab,ti. | 114 | student*.ab,ti. | 114 | student*.ab,ti. | 114 | student*.ab,ti. |
| 115 | schoolchild*.ab,ti. | 115 | schoolchild*.ab,ti. | 115 | schoolchild*.ab,ti. | 115 | schoolchild*.ab,ti. |
| 116 | preadolescen*.ab,ti. | 116 | preadolescen*.ab,ti. | 116 | preadolescen*.ab,ti. | 116 | preadolescen*.ab,ti. |
| 117 | minor*.ab,ti. | 117 | minor*.ab,ti. | 117 | minor*.ab,ti. | 117 | minor*.ab,ti. |
| 124 | 100-117 OR | 125 | 100-117 OR | 126 | 100-117 OR | 125 | 100-117 OR |
|  |  |  |  |  |  |  |  |
| 128 | 123 AND 124 AND 126 AND 127 | 129 | 124 AND 125 AND 127 AND 128 | 130 | 125 AND 126 AND128 AND 129 | 129 | 124 AND 125 AND 127 AND 128 |
